# The COSMIC (Catalogue of Somatic Mutations in Cancer) database and website

**DOI:** 10.1038/sj.bjc.6601894

**Published:** 2004-06-08

**Authors:** S Bamford, E Dawson, S Forbes, J Clements, R Pettett, A Dogan, A Flanagan, J Teague, P A Futreal, M R Stratton, R Wooster

**Affiliations:** 1 Wellcome Trust Sanger Institute, Wellcome Trust Genome Campus, Hinxton, Cambridgeshire CB10 1SA, UK; 2 Department of Histopathology, Royal Free and University Medical School, University Street, London WC1E 6JJ, UK; 3 The Institute of Orthopaedics, UCL, Stanmore, Middlesex HA7 4LP, UK

**Keywords:** somatic, mutation, database, website

## Abstract

The discovery of mutations in cancer genes has advanced our understanding of cancer. These results are dispersed across the scientific literature and with the availability of the human genome sequence will continue to accrue. The COSMIC (Catalogue of Somatic Mutations in Cancer) database and website have been developed to store somatic mutation data in a single location and display the data and other information related to human cancer. To populate this resource, data has currently been extracted from reports in the scientific literature for somatic mutations in four genes, BRAF, HRAS, KRAS2 and NRAS. At present, the database holds information on 66 634 samples and reports a total of 10 647 mutations. Through the web pages, these data can be queried, displayed as figures or tables and exported in a number of formats. COSMIC is an ongoing project that will continue to curate somatic mutation data and release it through the website.

Approximately one in three individuals in Europe and North America develops one of the approximately 200 different classes of cancer and it is the cause of death of one in five (Higginson, 1992). All cancers arise as a result of the acquisition of a series of fixed DNA sequence abnormalities, each of which ultimately confers growth advantage upon the clone of cells in which it has occurred (Vogelstein and Kinzler, 1998). These abnormalities include base substitutions, deletions, amplifications and rearrangements. The extent to which each of these mechanisms contributes to cancer varies markedly between different genes, and probably also between different cancer types. Identification of the genes that are mutated in cancer is a central aim of cancer research. Over the past 25 years, approximately 300 genes have been shown to be somatically mutated in cancer (Futreal *et al*, 2004). This work forms the foundation for understanding the biological abnormalities within neoplastic cells, provides information on the function of gene products and sheds light on more complex questions such as the relationships between genes and biochemical pathways. Current strategies for the development of new therapeutic and preventive agents in cancer are increasingly dependent upon modulation of these critical molecular targets.

The scientific literature is a rich source of mutation data that, in general, is published in a piecemeal fashion. More comprehensive data sources do exist, such as Online Mendelian Inheritance in Man (OMIM, Wheeler *et al*, 2004), HGVbase (Fredman *et al*, 2002) and the Human Gene Mutation Database (HGMD, Stenson *et al*, 2003). These databases give overviews of the genetics and biology of many genes and associated diseases (OMIM), genome variants and associated genotype–phenotype relationships (HGVbase) or germline mutation data (HGMD). For somatic mutations in cancer, there are many locus-specific web resources, such as those for p53 (Olivier *et al*, 2002; Béroud and Soussi, 2003), that cover a single gene in depth. The value of these various databases should not be underestimated; however, none of them offer a comprehensive view of all previously reported somatic mutations in cancer. Looking to the future, the volume of somatic mutation data will continue to expand and the scientific community will be better served if this data is provided in a coherent fashion. A public, comprehensive, intuitive, accessible and integrated database is required to maximise the benefit from this rich data set. The Catalogue of Somatic Mutations in Cancer (COSMIC), (http://www.sanger.ac.uk/cosmic) is a database that holds somatic mutation data and associated information, and can be interrogated through a series of web pages to provide a graphical or tabular view of the data along with various export options. To date, the database has been populated with data from four genes: HRAS, KRAS2, NRAS and BRAF.

## DATA CURATION

### Gene selection

The genes that have been selected for curation are taken from the list of cancer genes assembled in the Cancer Gene Census (Futreal *et al*, 2004). In the first instance, data was obtained for four genes that are known to be somatically mutated in cancer: HRAS (Reddy *et al*, 1982), KRAS2 (McCoy *et al*, 1983), NRAS (Hall *et al*, 1983) and BRAF (Davies *et al*, 2002).

### Data extraction from the literature

PubMed (Wheeler *et al*, 2004) is broadly searched for references containing relevant somatic mutation data in cancer (example search: (ras OR genes, ras) AND human AND mutation). In the first instance, the abstract is read to identify, and select for inclusion in the database, papers that are likely to include somatic mutation information relating to cancer or precancerous conditions. Primary research papers are read and information about the samples, mutations and experimental methods (see Table 1

**Table 1:** Data entered in COSMIC

**Reference**	**Sample**
Title	Gene
Authors	Experimental information
Journal	Sample ID
Year	Mutation status
Volume	Normal tissue tested
Page start and stop	Site primary
PubMed ID	Site subtype 1
Experimental information	Site subtype 2
Gene	Histology
	Histology subtype 1
**Mutation**	Histology subtype 2
Mutation ID	Stage
Mutation type	Grade
DNA location	Source tissue
DNA change	Loss of heterozygosity
DNA evidence	Gender
Is somatic	Age
RNA label	Other mutations
RNA change	Ethnicity
RNA region	Geographical location
RNA location	Parent tested
RNA evidence	Family ID
Amino-acid label	Remark
Amino-acid location	Reference
Amino-acid change	Environmental variables
Amino-acid evidence	
Gene	**Gene**
Sequence	Name
Remark	Symbol
	Other names
**Experimental information**	Chromosome
Primary detection method	Chromosome band
Secondary detection method	cDNA sequence accession
Confirmation method	cDNA sequence version
Exons/codons screened	Ensembl gene start and stop
Whole gene screened	Swissprot accession
Remark	OMIM accession

Section heading for the data in COSMIC are in bold.

) is extracted and entered into the database. Reviews are also selected if thought to be specific to a gene of interest. In order to avoid duplication of data, this source is used to identify the relevant primary literature and not as the source of the mutation data. Any references containing incomplete data (e.g. mutations reported but not fully described) or data of insufficient quality (e.g. errors identified in the data) are not fully curated but are added to a list of additional references containing somatic mutation information. Simple mutations are fed through Mutation Checker (Stajich *et al*, 2002) before being imported to COSMIC, while more complex alterations are manually annotated.

## COSMIC DATABASE

The COSMIC database is implemented in an Oracle relational database and has five sections each containing multiple tables.

### Gene information

A static version of each gene is maintained in COSMIC. The genomic structure of each gene and chromosome location is derived from Ensembl (Birney *et al*, 2004) and cDNA sequence and protein sequence from the RefSeq project (Wheeler *et al*, 2004). Other information is held to provide links to web resources such as Ensembl (Birney *et al*, 2004), Pfam (Bateman *et al*, 2004), InterPro (Mulder *et al*, 2003) and OMIM (Wheeler *et al*, 2004).

### Paper information

The details of the papers that have been curated are maintained in the paper section and include title, journal, author lists and links to PubMed. There are currently 1483 papers in COSMIC, 865 of these have been curated for mutations, while 618 either have no relevant data or incomplete data that could not accurately be extracted. By gene 30, 249, 718 and 303 papers report BRAF, HRAS, KRAS2 and NRAS mutations, respectively. Of the 865 papers reporting mutations, 615 report data on only one gene, while 72, 174 and four contain data on two, three or all four genes, respectively.

### Mutation information

COSMIC can accommodate information on base substitutions, insertions and deletions, translocations and changes in copy number. For the four genes presently in COSMIC, there are 147 unique mutations (36 for BRAF, 27 for HRAS, 52 for KRAS2 and 32 for NRAS). In the tumours that have been analysed, there are a total of 10 647 mutations, 736 in BRAF, 477 in HRAS, 8302 in KRAS2 and 1132 in NRAS.

### Tumour classification system

The tissue site and histology data is taken from the curated papers and entered into COSMIC (this forms the ‘paper definition’). Tumour classification is a continually evolving field and there is no standard nomenclature adhered to for the purposes of publication in the various journals. Identical tissues and histologies can have different labels depending on the origin and age of the study. To overcome difficulties caused by these alternate nomenclatures, a standardised system of definitions has been developed (the ‘COSMIC definitions’) through consultation with experts in the field. This groups data from the same tissue types and histologies and can be used to translate the ‘paper definitions’ to ‘COSMIC definitions’. Every sample has up to eight definitions; primary tissue, tissue subtype 1, 2 and 3, primary histology and histology subtypes 1, 2 and 3. If there is no data for any of these definitions, COSMIC records an entry of NS, not specified. A total of 513 tissue definitions have been noted in the papers in COSMIC and have been translated to 372 COSMIC tissue definitions. Likewise, a total of 1150 histology definitions were found in the papers in COSMIC that were translated to 425 COSMIC histology definitions. This unified classification system is presented through the web pages to present a normalised browsing tool.

### Individual/tumour/sample data

The sample data is taken from the curated papers and linked to the appropriate gene, paper, classification and when present a mutation. This forms the core of the COSMIC database. An individual can have many tumours and each tumour can have many samples. However in the COSMIC scheme, each sample is unique and could be considered as a single experiment. There are 66 634 sample records in COSMIC (5158, 11 876, 35 716 and 13 884 for BRAF, HRAS, KRAS2 and NRAS, respectively). These samples are derived from 57 444 tumours of which 51 988 were analysed in one gene, 2353 in two genes, 2930 in three genes and 173 in all four genes.

## COSMIC WEBSITE

**Figure 1:**
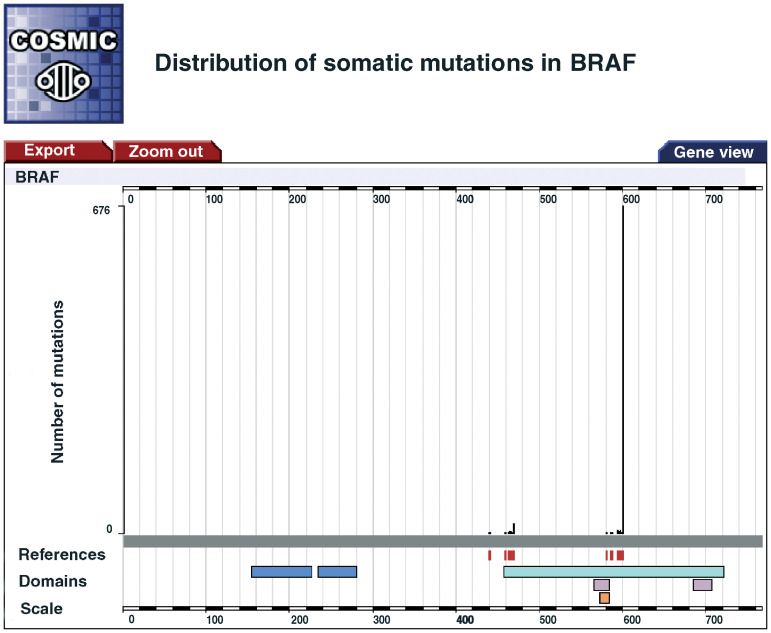
The initial output from COSMIC is a graphical view of the mutations distributed along the linear amino-acid sequence of the gene. The scale bar incorporates a zoom function to generate a more detailed view of the protein to the point where individual amino acids are named (when there are fewer than 31 amino acids displayed). When a Pfam or Interpro domain is present, a link is provided to these resources (adjacent to the Domain label) while links to the papers that were curated are positioned beneath the mutations (in red) with an option of either viewing the papers that have data for a particular location in the protein or all of the papers for the selected gene.

A series of web pages provides query tools to interrogate COSMIC and produces graphical (Figure 1

) and tabular (Table 2

**Table 2:** Mutation Details from COSMIC

	**Details for BRAF**		
**Tissue**	**Mutations (% of All Samples)**	**All Samples**	**Mutation Data**
NS	0	3	More Details
adrenal gland	0	2	More Details
autonomic ganglia	0	27	More Details
bile duct	16 (23%)	70	More Details
bladder	0	37	More Details
bone	1 (3%)	31	More Details
brain	4 (7%)	56	More Details
breast	1 (1%)	78	More Details
cervix	0	49	More Details
endometrium	0	5	More Details
eye	0	31	More Details
haematopoietic and lymphoid tissue	4 (1%)	322	More Details
head neck	6 (4%)	152	More Details
kidney	0	12	More Details
large intestine	148 (13%)	1135	More Details
larynx	0	25	More Details
liver	1 (3%)	32	More Details
lung	15 (2%)	829	More Details
mouth	0	13	More Details
ovary	57 (20%)	282	More Details
pancreas	5 (4%)	114	More Details
pharynx	3 (6%)	51	More Details
placenta	0	1	More Details
pleura	0	3	More Details
prostate	0	43	More Details
skin	282 (61%)	460	More Details
small intestine	0	1	More Details
soft tissue	5 (2%)	211	More Details
stomach	7 (2%)	407	More Details
testis	0	7	More Details
thyroid	181 (27%)	669	More Details

The mutations from COSMIC are presented by tissue and where selected by histology with a figure for the number of samples analysed for each tissue (All Samples) and the number of mutations reported (Mutated). The ‘More Details’ column gives further navigation options to view data for the selected tissue, view data for the same tissue in other genes or provide more details on the mutations for the selected tissue.

) displays of the data. Currently the output is provided at the amino-acid level based on the protein structure of each gene.

### Browse by gene

Immediate access to the data is provided through the *Browse by Gene* link. This gives an instant overview of the mutation data for one or more genes and gives links to display data for individual tissues.

### Browse by tissue

More complex queries can be constructed using the *Browse by Tissue* link. The user has the option to select one or more tissues, then one or more histologies, and finally one or more genes. If only one tissue or histology is selected, it is possible to select one or more tissue or histology subtypes before making a gene selection. All of the tissues present in the COSMIC classification scheme are available from the first page; however, subsequent pages only show the relevant options and not the entire list of options, for example having selected eye, the tissue subtype options are retina and uveal tract.

### Data display

After querying the database, the results are displayed as a figure (Figure 1) and as a series of tables (Table 2) for each gene that was selected. The figure shows the linear amino-acid sequence derived from the gene with the mutations positioned along its length. Further information and links are provided as appropriate to the protein sequence. The table gives a summary of the mutations stratified by tissue and histology. The depth of the stratification relates to the depth of the original query. If only tissue was selected, the data will be stratified by tissue; however, if tissue, subtissue, histology and subhistology are selected, the data will be broken down further. Links from this table reload the figure to display a subset of the data and provide more details of the specific mutations. Two other tables provide a summary of the statistics in COSMIC for the selected gene and a summary of the mutations shown in the figure.

### Exports and downloads

Having displayed the results from a query, the data can be formatted in simple text, Excel or HTML that can be downloaded from the COSMIC site. The cDNA and protein sequences are available through the *Additional Info*. link on the COSMIC home page as is the Classification Scheme.

## FUTURE DIRECTIONS

There is a continuing effort to enter additional somatic mutation data in to COSMIC. In order to keep the data in COSMIC up-to-date, we regularly monitor the literature for new reports of mutations in the genes that exist in COSMIC. In addition, further cancer genes will be taken from the Cancer Gene Census (Futreal *et al*, 2004) and curated. The COSMIC website will be developed further to make use of the underlying data. This will include a DNA view of the mutations and methods to display insertions and deletions. In addition, we will display other data that has already been captured such as the patient sex and age for the samples and the experimental methods used to screen for the mutations. There are however limitations to this data as we can only collect data that is described in the original work. Even with this caveat the data provides a direct summary of the somatic mutation literature. Considering the data set as a whole it will be possible to analyse, in greater detail, the wider aspects of the biology underlying the genetic changes that take place in cancer.

## References

[bib1] Bateman A, Coin L, Durbin R, Finn RD, Hollich V, Griffiths-Jones S, Khanna A, Marshall M, Moxon S, Sonnhammer EL, Studholme DJ, Yeats C, Eddy SR (2004) The Pfam protein families database. Nucleic Acids Res 32: D138–D141 14681378 10.1093/nar/gkh121 PMC308855

[bib2] Béroud C, Soussi T (2003) The UMD-p53 database: new mutations and analysis tools. Hum Mutat 21: 176–181 12619103 10.1002/humu.10187

[bib3] Birney E, Andrews D, Bevan P, Caccamo M, Cameron G, Chen Y, Clarke L, Coates G, Cox T, Cuff J, Curwen V, Cutts T, Down T, Durbin R, Eyras E, Fernandez-Suarez XM, Gane P, Gibbins B, Gilbert J, Hammond M, Hotz H, Iyer V, Kahari A, Jekosch K, Kasprzyk A, Keefe D, Keenan S, Lehvaslaiho H, McVicker G, Melsopp C, Meidl P, Mongin E, Pettett R, Potter S, Proctor G, Rae M, Searle S, Slater G, Smedley D, Smith J, Spooner W, Stabenau A, Stalker J, Storey R, Ureta-Vidal A, Woodwark C, Clamp M, Hubbard T (2004) Ensembl 2004. Nucleic Acids Res 32: D468–D470 14681459 10.1093/nar/gkh038 PMC308772

[bib4] Davies H, Bignell GR, Cox C, Stephens P, Edkins S, Clegg S, Teague J, Woffendin H, Garnett MJ, Bottomley W, Davis N, Dicks E, Ewing R, Floyd Y, Gray K, Hall S, Hawes R, Hughes J, Kosmidou V, Menzies A, Mould C, Parker A, Stevens C, Watt S, Hooper S, Wilson R, Jayatilake H, Gusterson BA, Cooper C, Shipley J, Hargrave D, Pritchard-Jones K, Maitland N, Chenevix-Trench G, Riggins GJ, Bigner DD, Palmieri G, Cossu A, Flanagan A, Nicholson A, Ho JW, Leung SY, Yuen ST, Weber BL, Seigler HF, Darrow TL, Paterson H, Marais R, Marshall CJ, Wooster R, Stratton MR, Futreal PA (2002) Mutations of the BRAF gene in human cancer. Nature 417: 949–954 12068308 10.1038/nature00766

[bib5] Fredman D, Siegfried M, Yuan YP, Bork P, Lehväslaiho H, Brookes AJ (2002) HGVbase: a human sequence variation database emphasizing data quality and a broad spectrum of data sources. Nucleic Acids Res 30: 387–391 11752345 10.1093/nar/30.1.387 PMC99093

[bib6] Futreal PA, Down T, Coin L, Marshall M, Rahman N, Wooster R, Timothy Hubbard T, Bateman A, Stratton MR (2004) A census of human cancer genes. Nat Rev Cancer 4: 177–183 14993899 10.1038/nrc1299 PMC2665285

[bib7] Hall A, Marshall CJ, Spurr NK, Weiss RA (1983) Identification of transforming gene in two human sarcoma cell lines as a new member of the ras gene family located on chromosome 1. Nature 303: 396–400 6304521 10.1038/303396a0

[bib8] Higginson J (1992) Human cancer: epidemiology and environmental causes. In: Higginson, Muis, Munoz (eds). Cambridge Monographs on Cancer Research. Cambridge, UK: Cambridge University Press

[bib9] McCoy MS, Toole JJ, Cunningham JM, Chang EH, Lowy DR, Weinberg RA (1983) Characterization of a human colon/lung carcinoma oncogene. Nature 302: 79–81 6298638 10.1038/302079a0

[bib10] Mulder NJ, Apweiler R, Attwood TK, Bairoch A, Barrell D, Bateman A, Binns D, Biswas M, Bradley P, Bork P, Bucher P, Copley RR, Courcelle E, Das U, Durbin R, Falquet L, Fleischmann W, Griffiths-Jones S, Haft D, Harte N, Hulo N, Kahn D, Kanapin A, Krestyaninova M, Lopez R, Letunic I, Lonsdale D, Silventoinen V, Orchard SE, Pagni M, Peyruc D, Ponting CP, Selengut JD, Servant F, Sigrist CJA, Vaughan R, Zdobnov EM (2003) The InterPro Database, 2003 brings increased coverage and new features. Nucleic Acids Res 31: 315–318 12520011 10.1093/nar/gkg046 PMC165493

[bib11] Olivier M, Eeles R, Hollstein M, Khan MA, Harris C.C, Hainaut P (2002) The IARC TP53 Database: new online mutation analysis and recommendations to users. Hum Mutat 19: 607–614 12007217 10.1002/humu.10081

[bib12] Reddy EP, Reynolds RK, Santos E, Barbacid M (1982) A point mutation is responsible for the acquisition of transforming properties by the T24 human bladder carcinoma oncogene. Nature 300: 149–152 7133135 10.1038/300149a0

[bib13] Stajich JE, Block D, Boulez K, Brenner SE, Chervitz SA, Dagdigian C, Fuellen G, Gilbert JG, Korf I, Lapp H, Lehvaslaiho H, Matsalla C, Mungall CJ, Osborne BI, Pocock MR, Schattner P, Senger M, Stein LD, Stupka E, Wilkinson MD, Birney E (2002) The Bioperl toolkit: Perl modules for the life sciences. Genome Res 12: 1611–1618 12368254 10.1101/gr.361602 PMC187536

[bib14] Stenson PD, Ball EV, Mort M, Phillips AD, Shiel JA, Thomas NS, Abeysinghe S, Krawczak M, Cooper DN (2003) Human Gene Mutation Database (HGMD(R)): 2003 update. Hum Mutat 21: 577–581 12754702 10.1002/humu.10212

[bib15] Vogelstein B, Kinzler K (1998) The Genetic Basis of Human Cancer. New York: McGraw Hill

[bib16] Wheeler DL, Church DM, Edgar R, Federhen S, Helmberg W, Madden TL, Pontius JU, Schuler GD, Schriml LM, Sequeira E, Suzek TO, Tatusova TA, Wagner L (2004) Database resources of the National Center for Biotechnology Information: update. Nucleic Acids Res 32: D35–D40 14681353 10.1093/nar/gkh073 PMC308807

